# Carriage rate of *Neisseria meningitidis*, antibiotic susceptibility pattern and associated risk factors among primary school children in Gondar town, Northwest Ethiopia

**DOI:** 10.1186/s12879-020-05080-w

**Published:** 2020-05-20

**Authors:** Zelalem Tefera, Feleke Mekonnen, Moges Tiruneh, Teshome Belachew

**Affiliations:** 1 Kemissie General Hospital, Kemise, Northeast Ethiopia; 2 Department of Medical Microbiology, College of Medicine and Health Sciences, Bahir Dar University, Bahir Dar, Ethiopia; 3 Department of Medical Microbiology, School of Biomedical and Laboratory Sciences, College of Medicine and Health Sciences, University of Gondar, Gondar, Ethiopia

**Keywords:** Carriage, *N. meningitidis*, School children, Antimicrobial susceptibility

## Abstract

**Background:**

Globally, in 2012, about 1.2 million estimated cases were reported with ~ 135,000 deaths annually. In Ethiopia, specifically in our study area, limited information is found on the oropharyngeal carriage, antimicrobial resistance pattern, and associated risk factors for *N. meningitidis* among school children. So, the aim of this study was to assess oropharyngeal carriage rate of *N. meningitidis,* antibiotic susceptibility pattern and associated risk factors among primary school children in Gondar town, Northwest Ethiopia.

**Methods:**

A cross sectional study was conducted from January to April, 2019 in Gondar town. Multi stage simple random sampling technique was used. A total of 524 oropharyngeal swabs were collected using sterile plastic cotton swabs. Modified Thayer Martin media was used for primary isolation. Antimicrobial susceptibility pattern was done based on Kirby-Bauer method on Muller-Hinton agar supplemented with 5% sheep blood. Multidrug resistance was defined as resistance of an isolate to two or more antimicrobial classes tested. Logistic regression model was used to see the association between dependent variables (Carriage rate of *Neisseria meningitidis*, Serogroups of *Neisseria meningitidis and* Antimicrobial susceptibility patterns) and independent variables (Socio-demographic data and risk factors). Variables with a *P*- value ≤0.2 during bivariable analysis was taken to multivariable analysis to check significant association of meningococcal carriage with risk factors. Finally, a *P*-value < 0.05 was considered as statistically significant. Data was summarized using numbers, percentages and tables.

**Results:**

A total of 53(10.1%) (CI: 7.6–12.8) *N. meningitidis* isolates were identified. Serogroup A 13 (24.5%) was the most prevalent followed by Y/W135 11(20.7%) whereas serogroup B 4(7.6%) was the least identified serotype. Meningococcal isolates were resistant to ciprofloxacin (45.3%) and trimethoprim-sulfamethoxazole (73.6%). Overall, most of meningococcal isolates showed about 32(60.4%) multidrug resistance. Meningococcal carriage rate was significantly associated with family size, tonsillectomy, passive smoking, number of students per class, sharing utensils, history of visiting healthcare institutions, and indoor kitchen.

**Conclusion:**

This study highlights the need for reinforcement of case-based, laboratory confirmed surveillance of *N. meningitidis* carriage in Ethiopian elementary school students to enable mapping of distribution of serotypes of the causative organisms across the country and determine the current potential necessity of vaccination.

## Background

*Neisseria meningitidis* is a Gram-negative diplococci with 13 distinct serotypes. It inhabits the mucosal surface of nasopharynx and oropharynx [1]. However, about 90% of human disease are caused by serogroup A, B, C, X, Y, and W135 [2]. About 5–10% of healthy people carry *N. meningitidis* in the nasopharynx and oropharynx and during epidemics, the carrier state rises to 70–80% [3]. Different factors can enhance carriage rate: Immunological susceptibility, travel, large population displacement, poor living condition, overcrowding, housing condition and climatic condition [4].

Meningococcal infection has been a big threat for the globe and exists as sporadic, hyper-sporadic, and epidemic disease. In 2012, an estimated 1.2 million cases of meningococcal infection per year was reported, with ~ 135,000 deaths worldwide [5]. The African meningitis belt is more affected area of bacterial meningitis characterized by distinct seasonal patterns. The disease incidence peaks in the dry season [6]. In Ethiopia, a major epidemic was recorded in 2001with 6964 cases and 330 deaths. Another epidemic was also recorded in 2003–2004 with 3326 cases and 160 deaths [7]. In 2017, a study was done in Addis Ababa, Ethiopia among school children and 20.4% of *N. meningitidis* carriage was documented [8].

Meningococcal infection can be prevented through taking different measures and vaccination can play a pivotal role. As the result, in 2010, scientists developed the new conjugate vaccine, MenAfrivac vaccine, and implemented in sub-Saharan Africa. This vaccine is developed from, *N. meningitidis* serogroup A capsular polysaccharide and vaccination was implemented among 1–29 years old individuals [9]. Mass vaccination campaign with the same vaccine was also practiced in Ethiopia, and vaccination has been implemented in 3 phases from 2013 to 2015 [10]. In the study area, limited information is found on the oropharyngeal carriage, antimicrobial resistance pattern and associated risk factors for *N. meningitidis* especially in school children. Thus, carriage studies are important to improve our understanding of the *N. meningitidis* serogroup distribution and also the epidemiology of meningococcal disease control. Thus, it is important to determine the percentage of carriage rates. If the rate of carriers were identified, then tools to reduce personal contacts could be provided in populations with a high carrier rate. This process may include avoidance of crowding, reconstruction of the air-condition systems of the dorms, personal health education, or the administration of vaccines. So, the aim of this study was to assess oropharyngeal carriage rate of *N. meningitidis,* antibiotic susceptibility pattern and associated risk factors among primary school children in Gondar town, Northwest Ethiopia.

## Methods

### Study setting, design, and period

A community-based cross-sectional study was conducted among primary school children in Gondar town, North West Ethiopia, from January to April 2019. This study was conducted in six primary schools. Gondar is found 737 km from Addis Ababa, the capital city of Ethiopia, and 180 km from Bahir Dar, the capital of Amhara national regional state. Gondar town and its surroundings have 44 elementary schools, 11 secondary schools and 30 kindergartens.

### Sample size and sampling technique

The sample size (524) was determined by using a single population proportion formula by considering the prevalence of 20.4% [8], with a 95% confidence interval, and a 5% margin of error, with 10% no-response rate and design effect. The multistage sampling technique was used to select schools. Then schools were stratified to grades and sections. The total number of study participants were allocated proportionally to each school, grades and sections based on the school sampling frame and the study subjects were selected by simple random sampling technique (Table 1).

**Table 1:** List of selected elementary schools and number of selected students in Gondar town, Northwest Ethiopia, January to April, 2019

S.no	Elementary school	Students per school	Proportion	Proportionally allocated number of students per school	Number of students taken (sample)
**1**	Abiwotfire	2010	23.4	128	120
**2**	Hubret	1090	12.7	70	66
**3**	AtseBekafa	1146	13.2	73	64
**4**	TsadikuYohanis	1450	16.8	92	92
**5**	Meseret	1512	17.6	97	94
**6**	Chechela	1400	16.3	90	88
**Total**		8608	100	550	524

### Data collection and laboratory methods

#### Data collection procedures

A pre-tested questionnaire based on postulated or known risk factors was developed and modified to explore the objectives of the study. Then, it was checked on school children who were not included in the study. It was prepared in English and translated to Amharic then translated back into English to check the accuracy of the translation. The questionnaire design included two parts; socio-demographic characteristics and associated risk factors.

The questionnaire and assent/consent form were distributed to the selected students at school after informing the purpose of the study and the right of the study participants. Questionnaire and assent/consent form were also distributed to guardians and emphasis was given to return the questionnaire and assent/consent form after 24 h. Students living alone (with the age range of 17–18 years) were considered as adults and informed to fill the questionnaire and sign the consent form at school. Socio-demographic characteristics and other relevant information filled by the parents/guardians and students were collected at school by trained laboratory technologists before sample collection.

### Laboratory methods

#### Oropharyngeal sample collection

Oropharyngeal swabs were collected by a trained medical microbiologist using a plain cotton swab (Unison Narula, India) using tongue depressor (Unison Narula group, India) at the posterior pharyngeal wall behind the uvula and tonsils of each volunteer participant. After collection, samples were transported by using Amies transport media (Bio mark, India) to the University of Gondar teaching hospital laboratory within 2 h of collection within a cold box.

#### Culture and identification

Once the specimens reached to Gondar University teaching laboratory, it was inoculated on Modified Thayer Martin (MTM) culture media (Oxoid, UK). The inoculated MTM plates were incubated at 37 °C with 5–10% CO_2_ for 24 to 48 h. A presumptive diagnosis was done by gram stain and colony characteristics on the agar plate. Medium to large, round, smooth, convex, colorless-to-grey, opaque colonies on the MTM was further confirmed by the oxidase test (Deben Diagnostics Ltd., UK). After confirmation, the presence of gram-negative diplococcus with oxidase-positive, isolates were sub-cultured on a blood agar plate (BAP) (Oxoid, UK) with 5–10% CO_2_ for 24 to 48 h, to guarantee the purity of colonies for the biochemical test. Plates were monitored every 24 h for the growth of typical colonies.

Carbohydrate utilization test (glucose, maltose, lactose, and sucrose) was performed by cystine trypticase agar (CTA) (SRL, India) to further differentiate *Neisseria meningitidis* from *Moraxella* species and other nonpathogenic *Neisseria* species. Isolates with gram-negative diplococci, oxidase-positive, glucose fermenter, maltose fermenter, lactose and sucrose none- fermenter were interpreted and confirmed as *N. meningitidis*. Once the species are known, the serogroup of isolates was determined with the slide agglutination method while using commercially prepared antiserum A, B, C, W135/Y, and X (Bio-Rad, France) and antiserum X (BD Difco, USA). Negative for these six serogroups was classified as non-serogroupable [8].

#### Antimicrobial susceptibility testing

Antimicrobial susceptibility testing was carried out on isolates of *N. meningitidis* by using disc diffusion technique as per the standard Kirby-Bauer method on Mueller-Hinton agar (Bio mark, India) supplemented with 5% sheep blood at 37°c for 18–24 h [11]. A suspension of the test organism was prepared equivalent to 0.5 McFarland. The surface of Mueller-Hinton agar supplemented with 5% sheep blood was completely covered by rotating the swab. The plates were allowed to dry for 3–5 min; then discs were evenly distributed on the inoculated plate using sterile forceps and incubated in 5–10% CO_2_ at 37°c for 20–24 h. The following routinely used antimicrobial agents were tested: cefotaxime (30 μg), minocycline (30 μg) meropenem (10 μg), azithromycin (15 μg), ciprofloxacin (5 μg), trimethoprim-sulfamethoxazole (1.25/ 23.75 μg), chloramphenicol (30 μg), and rifampin (5 μg). Diameters of the zone of inhibition around the disc was measured to the nearest millimeter using a graduated caliper in millimeters and results were classified as sensitive, intermediate and resistant based on CLSI-2018 guideline [12]. Multidrug resistance was defined as resistance of an isolate to two or more antimicrobial classes tested [13].

#### Laboratory data quality assurance

Preanalytical, analytical and post analytical quality assurance was maintained [14].

#### Data analysis and interpretation

All data was entered to EPI info version 7 for data clearance and consistency and exported to SPSS version 20.0 for analysis. Descriptive statistics was computed to calculate frequencies. The magnitude of the association between different variables and oropharyngeal meningococcal carriage was assessed using bivariate and multivariate analysis. Variables which had a *P*- value ≤0.20 for bivariate analysis was taken to multivariate analysis to check real association of meningococcal carriage rate with risk factors and expressed by adjusted odds ratio at 95% confidence interval. A *P*-value < 0.05 was considered as statistically significant. Data was summarized using numbers, percentages and tables.

#### Ethical considerations

The study was conducted after obtaining institutional ethical clearance (“Ref No-SBMLS/2123/11”) from University of Gondar. Support letter was sought from Gondar town educational office. Assent from the parents/guardians of youth students and assent/consent from the study participants was obtained.

## Results

### Demographic characteristics of study participants

A total of 524 school children (283 males and 241 females) were included in this study. The mean ± SD age of the participants was 12.2 ± 2.74 years. About 49% of the study participants were within the age group of 11–14 years (Table 2).

**Table 2:** Socio-demographic characteristics of all participants among primary school children in Gondar town, Northwest Ethiopia, January to April 2019

Characteristics of children (***n*** = 524)	Frequency	%
**Sex**		
Male	283	54%
Female	241	46%
**Age**		
7–10	148	28.2%
11–14	257	49%
15–18	119	22.7%
**Religion**		
Orthodox	419	80.0%
Muslim	100	19.1%
Protestant	2	0.4%
Catholic	1	0.2%
Other	2	0.4%
**Residence**		
Rural	10	1.9%
Urban	514	98.1%
**Grade level**		
1–4	253	48.3%
5–8	271	51.7%

### Oropharyngeal carriage isolates

The overall prevalence of *Neisseria meningitidis* was 53(10.1%) (95% CI: 7.6, 12.8). Meningococcal carriage identified among male 30/53 (56.6%) was higher than females 23/53 (43.4%) (Table 3).

**Table 3:** Distribution of oropharyngeal isolates by age, sex and school among primary school children in Gondar town, Northwest Ethiopia, January to April 2019

School	Age	*N. meningitides* (*N* = 53)	
		M	F
Abiwot fire	7–10	1	0
	11–14	3	0
	15–18	6	0
Hibret	7–10	0	1
	11–14	1	2
	15–18	0	3
AtseBekafa	7–10	0	1
	11–14	0	4
	15–18	0	0
TsadikuYohanis	7–10	5	4
	11–14	1	5
	15–18	0	0
Meseret	7–10	1	0
	11–14	7	0
	15–18	3	0
Chechela	7–10	0	0
	11–14	2	2
	15–18	0	1
Total n (%)		30 (56.6)	23 (43.4)

### Serogroup distribution of *N. meningitidis*

All types of invasive meningococcal serogroups were identified, of which, serogroup A was the leading isolate with the isolation rate of 13 (24.5%) followed by serogroup Y/W135, 11(20.7%). Serogroup B, 4 (7.5%) was the least identified isolate. Serogroup A dominates on male (15.1%) than female (9.4%) (Table 4).

**Table 4:** Serogroup distribution of *N. meningitidis* isolates by age and sex among primary school children in Gondar town, Northwest Ethiopia, January to April 2019

Age	Serogroups (***N*** = 53)												Total n (%)
	A(*n* = 13)		B(*n* = 4)		C(*n* = 8)		Y/W135 = 11		X(*n* = 5)		NG(*n* = 12)		
	M	F	M	F	M	F	M	F	M	F	M	F	
7–10	0	2	2	0	2	0	1	1	1	2	1	1	13 (24.5)
11–14	6	3	1	1	2	3	2	3	0	0	3	3	27 (50.9)
15–18	2	0	0	0	1	0	2	2	1	1	3	1	13 (24.5)
Total (%)	8 (15.1)	5 (9.4)	3 (5.7)	1 (1.9)	5 (9.4)	3 (5.7	5 (9.4	6 (11.3)	2 (3.8)	3 (5.7)	7 (13.2)	5 (9.4)	53 (100)

### Antimicrobial susceptibility patterns of *N. meningitidis*

*Neisseria. meningitidis* isolates were tested against routinely used antimicrobial agents. In this study, most of the meningococci isolates showed a high level of resistance to trimethoprim/ sulfamethoxazole (73.6%), ciprofloxacin (45.3%) and cefotaxime (35.8%). However, the majority of the isolates were susceptible to azithromycin (96.2%), chloramphenicol (92.5%) and minocycline (88.7%) (Table 5).

**Table 5:** Antimicrobial susceptibility patterns of meningococcal isolates among primary school children in Gondar town, Northwest Ethiopia, January to April 2019

Antimicrobials	Sensitivity	*N. meningitidis* (*n* = 53)						
		A = 13	B = 4	C = 8	W/Y = 11	X = 5	NG = 12	Total (=53) n (%)
Cefotaxime	S	8	1	7	5	3	10	34 (64.2)
	R	5	3	1	6	2	2	19 (35.8)
Minocycline	S	9	3	8	10	5	12	47 (88.7)
	R	4	1		1			6 (11.3)
Meropenem	S	10	3	8	9	5	10	45 (84.9)
	R	3	1		2		2	8 (15.1)
Azithromycin	S	12	4	8	10	5	12	51 (96.2)
	R	1			1			2 (3.8)
Ciprofloxacin	S	7	2	6	4	2	7	28 (52.8)
	I				1			1 (1.9)
	R	6	2	2	6	3	5	24 (45.3)
Trimethoprim/ sulfamethoxazole	S	1		1	3		5	10 (18.9)
	I	1				1	2	4 (7.5)
	R	11	4	7	8	4	5	39 (73.6)
Chloramphenicol	S	13	3	8	8	5	12	49 (92.5)
	I				1			1 (1.9)
	R		1		2			3 (5.7)
Rifampin	S	8	2	7	8	5	9	39 (73.6)
	I	2		1			1	4 (7.5)
	R	3	2		2		2	9 (17)

*A* Serogrup A, *B* Serogroup B, *C* Serogroup C, *W/Y* Serogroup W/Y, *X* Serogroup X, *NG* Non-serogroupable, *S* Sensitive, *R* Resistance, *I* Intermediate

### Multidrug resistance pattern of *N. meningitidis*

Multidrug resistance pattern of *N. meningitis* isolates was also determined. Overall, most of the meningococcal isolates showed a high level of multidrug resistance with the rate of 32(60.4%). On the serogroup level, serogroup B was 100% MDR followed by serogroup X, 80% and serogroup Y/W-135, 72.7%. Only 9 (16.9%) of the meningococcal isolates had no resistance for all class of antimicrobials tested. Similarly, about 50% of non-serogroupable (NG) isolates had no resistance to the tested class of antimicrobials (Table 6).

**Table 6:** Multi-drug resistance pattern for *Neisseria meningitidis* isolates among primary school children in Gondar town, Northwest Ethiopia, January to April 2019

Serogroup	Anti-microbial sensitivity pattern						Total	MDR ≥ 2 class
	R0	R1	R2	R3	R4	>R5		n/N (%)
A	1	3	2	3	3	1	13	9/13 (69.2)
B	0	0	2	1	0	1	4	4/4 (100)
C	1	5	1	1	0	0	8	2/8 (25)
Y/W135	1	2	3	2	1	2	11	8/11 (72.7)
X	0	1	4	0	0	0	5	4/5 (80)
NG	6	1	1	3	1	0	12	5/12 (41.7)
Total	9	12	13	10	5	4	53	32/53 (60.4)
**Percentile**	**16.9**	**22.7**	**24.5**	**18.9**	**9.4**	**7.6**	**100**	**60.4**

*R0* No resistance for any class of antimicrobial, *R1* Resistance for one class of antimicrobials, *R2* Resistance for two class of antimicrobial, *R3* Resistance for three class of antimicrobials, *R4* Resistance for four class of antimicrobials *R > 5* Resistance for five class of antimicrobials

### Associated risk factors of study participants

In this study, the average family size of students was 5.3 people per household and the average number of rooms per household was 2.3. From all study participants, 21% had a history of hospitalization at least for 1 day at health institutions. Of the total participants, the majority of (60.5%) had a history of tonsillectomy. Among the study participants who had a history of treatment before 2 weeks of the study period, 19.3% had poor treatment adherence. About 10.1% of the family of the study participants smokes cigarettes while 29.2% of the family had a history of living in crowded area (Table 7).

**Table 7:** Bivariate and multivariate analysis of risk factors for oropharnygeal carriage among primary school children in Gondar town, Northwest Ethiopia, January to April 2019

Variables	*N. meningitidis*		COR (95% C.I.)	AOR (95% C.I.)	*P*-value
	Yes n(%)	No n(%)			
Sex					
Male	30 (10.6)	253 (89.4)	1.12 (0.634–1.99)	_	_
Female	23 (9.5)	218 (90.5)	1	_	_
Age					
7–10	13 (8.8)	135 (91.2)	1.274 (0.567–2.86)	_	_
11–14	27 (10.5)	230 (89.5)	1.045 (0.519–2.105)	_	_
15–18	13 (10.9)	106 (89.1)	1	_	_
Grade level					
1–4	24 (9.5)	229 (90.5)	0.875 (0.494–1.55)	_	_
5–8	29 (10.7)	242 (89.3)	1	_	_
Family size					
> 5	29 (13.9)	180 (86.1)	1.953 (1.103–3.46)	2.71 (1.41–5.18)	0.003
≤5	24 (7.6)	291 (92.4)	1	1	
Number of beds/ houses					
Only one	42 (10.5)	360 (89.6)	1.18 (0.586–2.364)	_	_
> one	11 (9)	111 (91)	1	_	_
Sharing utensils					
Yes	48 (12.1)	349 (87.9)	3.356 (1.31–8.62)	4.15 (1.49–11.58)	0.007
No	5 (3.9)	122 (96.1)	1	1	
History of family visiting crowded area					
Yes	16 (10.5)	137 (89.5)	1.05 (0.568–1.958)	_	_
No	37 (9.97)	334 (90.03)	1	_	_
Visiting healthcare institutions					
Yes	46 (11.4)	358 (88.6)	2.07 (0.911–4.723)	2.76 (1.098–6.94)	0.031
No	7 (5.8)	113 (94.2)	1	_	_
Hospitalization					
Yes	14 (12.7)	96 (87.3)	1.40 (0.732–2.687)	_	_
No	39 (9.4)	375 (90.6)	1	_	_
Antibiotics adherence					
Yes	39 (12.1)	384 (87.9)	1	_	_
No	14 (13.9)	87 (86.1)	1.584 (0.824–3.05)	_	_
Tonsillectomy					
Yes	40 (12.6)	277 (87.4)	2.155 (1.123–4.136)	2.84 (1.36–5.93)	0.006
No	13 (6.3)	194 (93.7)	1	1	
Kitchen location					
Indoor	50 (11.7)	379 (88.3)	4.046 (1.234–13.26)	5.55 (1.53–20.17)	0.009
Outdoor	3 (3.2)	92 (96.8)	1	1	
Passive smoker					
Yes	8 (15.1)	45 (84.9)	1.68 (1.25–6.82)	4.62 (1.65–12.89)	0.004
No	45 (9.6)	426 (90.4)	1	1	
Number of students per class					
≤40	1 (1.7)	57 (98.3)	1	1	
> 40	52 (11.2)	414 (88.8)	7.159 (0.971–52.79)	7.81 (1.02–59.78)	0.048

### Risk factors analysis for oropharyngeal carriage of *N. meningitidis*

In bivariable logistic regression analysis associated factors with *P*-value < 0.2 were transferred to multivariable logistic regression to the significant association of these factors. Multivariable logistic statistical analysis showed that *N. meningitidis* oropharyngeal carriage had a significant association with family size (Adjusted Odds Ratio (AOR; 2.71 95% CI 1.41–5.18, *P* = 0.003)), sharing utensils (AOR; 4.15, 95% CI 1.49–11.58, *P* = 0.007), attending healthcare institutions (AOR;2.76, 95% CI 1.098–6.94, *P* = 0.031), history of tonsillectomy (AOR;2.84, 95% CI 1.36–5.93, *P* = 0.006), indoor kitchen (AOR;5.55, 95% CI, 1.53–20.17 *P* = 0.009), parental cigarette smoking (AOR;4.62, 95% CI,1.65–12.89, *P* = 0.004) and number of students per classroom (AOR;7.81, 95% CI, 1.02 59.78, *P* = 0.048) (Table 7).

## Discussion

Invasive meningococcal infection is a global problem occurring as sporadic, hyper-sporadic, and epidemic disease [15]. The problem has mainly occurred in the developing world especially in the African meningitis belt [16, 17]. In Ethiopia, meningitis outbreaks have been occurred over several years, being responsible for morbidity and mortality [7]. Many researched evidences showed that people who are carriers of *N. meningitidis* are at high risk of developing invasive meningococcal disease in their life time specifically, if they are exposed to factors that wanes the immune system. However, in Ethiopia, there is only limited information regarding the *N. meningitidis* carriage rate, antimicrobial susceptibility pattern and associated factors [4, 18]. Therefore, this study was intended to show the gap and fill the limited information on the oropharyngeal carriage, antimicrobial resistance pattern and associated risk factors for *N. meningitidis* especially in school children in the study area.

The overall *N. meningitidis* oropharyngeal carriage rate in this study was 10.1%. The predominant serogroup in our study was serogroup A (24.5%) and W135/Y (20.6%) while the least was serogroup B (7.6%). Despite the fact that menA vaccine mass vaccination campaign was implemented in 2012 in the study area, now it is 6 years after implementation and the prevalence of *N. meningitidis* serotype A will be reverted to high. The high prevalence of serotype W135/Y may be due to suppression of serotype A by the vaccine which in turn let serotype W135/Y to compete with other least prevalent serotypes. This overall carriage prevalence was markedly higher than a study conducted at Gondar University teaching hospital in 2012 (234 oropharyngeal swabs) among < 10 years OPD patients with 6% carriage [19]. The variation may result from the difference in the target population (asymptomatic vs symptomatic), time of investigation and sample size.

Our study also had a high prevalence of carriage than the studies conducted in Arba Minch, Southern Ethiopia among 7479 oropharyngeal samples with 6.6% prevalence [20] and in Gurage Zone, Southern Ethiopia with 4.6% carriage rate [21]. Surveillance of invasive meningitis isolates in Ethiopia in 2012–2013 showed that in Hawassa, in the southern part of the country, serogroup A was the dominant cause of disease [22]. But in 2015, menA vaccine was given and this may be the reason for the decrement of the carriage rate. On those mentioned studies, no serogroup A was identified and serogroup B was the least identified. Implementation of menA mass vaccination campaign may be the reason for zero prevalence of serotype A.

In contrast, our study had less carriage rate compared to three local studies conducted at Gondar university hospital (2019 CSF samples) in the year 2011 to 2013 with 18.4% prevalence [23], Addis Ababa (240 nasal swabs) with 20.4% carriage rate [8] and bacterial meningitis surveillance in Ethiopia, 2012–2013 (139 CSF samples) with 19.4% prevalence rate [24].

The antimicrobial susceptibility pattern of *N. meningitidis* was determined. In the present study, higher resistance was reported for cefotaxime (35.8%), ciprofloxacin (45.3%) and trimethoprim-sulfamethoxazole (73.6%). The increment of resistance may be due to the easy accessibility of drugs, the simplicity of taking drugs (oral route of administration) and the use of these antibiotics for a long period of time in the country especially ciprofloxacin and trimethoprim-sulfamethoxazole and on top of that, irrational drug use. Many studies done in developed countries showed that resistance to cefotaxime is rare. But, in the Southern parts of Ethiopia about 14% of cefotaxime resistance was reported which supports our findings [21].

Ciprofloxacin was another antimicrobial agent tested against N. meningitidis and high level of resistance (45%). In contrary to our study, in Addis Ababa [8] and Gondar [19], *N. meningitidis* was susceptible to ciprofloxacin with the rate of 83.7 and 78.6% respectively. This discrepancy might be due to the difference in antimicrobial usage practices.

In our study, associated risk factors like tonsillectomy (*P* = 0.006), large family size (*P* = 0.003), history of visiting health care institutions (*P* = 0.031), number of students per class greater than 40 (*P* = 0.048), indoor kitchen location (*P* = 0.009), sharing utensils (*P* = 0.007), and cigarette smoking (*P* = 0.004) were significantly associated with *N. meningitidis* carriage. In different studies determinants like family size [25], crowded living condition [8, 26], the number of children per house [27], the number of positive household members [28], lower socioeconomic status [29], indoor kitchen [26], and overcrowding in the house [30] were significantly associated risk factors in which coincided with our study finding.

## Conclusion

*Neisseria meningitidis* prevalence in the present study had a high carriage rate among males than females. Serogroup A and Y/W135 were predominantly circulating meningococcal isolates in the community. Meningococcal carriage rate among primary school students was significantly associated with larger family size, students with tonsillectomy, parental cigarette smoking, students with greater than 40 per class, sharing utensils, history of visiting healthcare institutions and indoor kitchen.

The antibiotics markedly resisted by meningococcal isolates were trimethoprim-sulfamethoxazole, ciprofloxacin, and cefotaxime. The effective antibiotics identified in this study were minocycline, azithromycin, meropenem and chloramphenicol. Most of the meningococcal isolates were identified as multidrug resistance, with serogroup B and serogroup X had markedly higher resistance. We recommend the scientific community as well as the health sector to perform continuous surveillance of *N. meningitidis* carriage to control any possible diversity and emerging virulent strains in high-risk populations as well as to predict the epidemiology of meningococcal infections and the clinical spectrum of affected populations. Especially, molecular identification is essential for identification of which genotypes is circulating. Health education should be strengthened to reduce *N. meningitidis* carriage and possible risk factors. Moreover, antibiotic stewardship should be well strengthened at all health facility level to reduce the expanding of drug resistance problem.

## Data Availability

The datasets used and/or analyzed during the current study are available from the corresponding author on reasonable request.

## Abbreviations

ATCC: American Type Culture Collection
BAP: Blood Agar Plate
CLSI: Clinical Laboratory Standard Institute
CSF: Cerebro Spinal Fluid
CTA: Cysteine Trypticase Agar
ELISA: Enzyme Linked Immuno-Sorbent Assay
MTM: Modified Thayer-Martín Media
OPD: Out-Patient Department
PCR: Polymerase Chain Reaction
SBML: School of Biomedical and Laboratory Science
